# Retrobulbar Hemodynamic Effects of Nipradilol in Normal and Normal-Tension Glaucoma Eyes

**DOI:** 10.1155/2011/652904

**Published:** 2011-11-03

**Authors:** Mitsushi Fukukita, Masashi Ido, Syunsuke Osawa, Mikio Sasoh, Motoyasu Furuta, Kunio Ito, Masahiko Sugimoto, Yukitaka Uji

**Affiliations:** Department of Ophthalmology, Mie University School of Medicine, Mie 514-8507, Japan

## Abstract

*Purpose*. To investigate the effects of nipradilol on retrobulbar hemodynamics. *Methods*. We investigated normal and normal-tension glaucoma (NTG) eyes. Topical nipradilol (one eye) and placebo eye drops (fellow eye) were instilled for 1 week in volunteers. Nipradilol was also instilled in NTG patients. Ultrasound color Doppler imaging for the posterior vessels was performed before, 2 hr, 1 week (for normal), and at 4 weeks (for NTG). *Results*. In normal eyes, there were significant decreases in the resistance index (RI) for the temporal short posterior ciliary arteries (PCA) at 2 hr and for the ophthalmic arteries at 1 week. There were no significant changes in the placebo-treated eyes. In the NTG eyes, there was a significant decrease in the RI for the central retinal artery, nasal, and temporal PCA at 2 hr and 4 weeks. *Conclusion*. Short-term observations found that nipradilol increased the ocular blood flow in normal and NTG eyes.

## 1. Introduction

Reduction of intraocular pressure (IOP) is one of the keys to preventing glaucoma progression. In some normal-tension glaucoma (NTG) patients, however, a visual field loss progresses despite achieving a sufficient reduction of the IOP [1]. Other factors that may be involved in this progression include frequency of migraine, optic disc hemorrhages, and low blood pressure. Many reports have proposed that there is a relationship between NTG and circulatory disorders or pathogenesis of the glaucomatous optic nerve change [2–4].

 To clarify the pathology in order to design new therapies, it is necessary to obtain a better understanding of the physiology of the circulatory dynamics in NTG eyes. As such, several reports have brought up the importance of retrobulbar hemodynamics in NTG. Short posterior ciliary arteries separate from the posterior ciliary arteries and then penetrate the eye wall around the insertion of the optic nerve head. These are essential for supplying blood to the optic nerve head [5]. In addition, these vessels also play an important role in the ocular blood circulation. To evaluate this ocular circulation, ultrasonic color Doppler imaging (CDI) is a very useful tool [6].

 Nipradilol (3,4-dehydro-8-(2-hydroxy-3-isopropylamino)propoxy-3-nitroxy-2H-1-bezeopyran) is a newly developed antiglaucoma ophthalmic agent, that is, a nonselective *β*-blocker with selective *α*-blocking activities [7–9]. Topical instillation of 0.25% nipradilol reduces the IOP and protects the visual field in patients with NTG, similar to that seen for timolol [10, 11]. Nipradilol differs from other antiglaucoma eye drops in that it induces vasodilatory action. In addition, nipradilol is able to rapidly reach the retrobulbar tissue at pharmacologic effective concentrations [12]. However, it is still not clear as to how it is able to exert its antiglaucomatous function within this region. 

 In the present study, we used the CDI system to examine the effect of topical 0.25% nipradilol administration on blood flow in various posterior ocular vessels in normal and NTG eyes.

## 2. Subjects and Methods

### 2.1. Subject Demographics

All procedures were approved by the Ethics Review Committee of the Mie University Hospital and followed the tenets of the Declaration of Helsinki. All subjects provided informed consent prior to initiation of the study.

 In the first part of the study, to evaluate whether nipradilol cause changes ocular blood flow on normal eyes, a total of 13 healthy volunteers with no ocular or systemic medications (normal group) were evaluated. The aim of this first study is to see its tendency, so we scheduled during one week to avoid side effect on subjects. On the first experimental day, topical 0.25% nipradilol (Hypadil Kowa Ophthalmic Solution, Kowa Inc., Tokyo, Japan) was instilled in one eye, while a placebo that was identical to the vehicle solution of the nipradilol eye drop and which was provided with coded labels (kind gift from Kowa Inc., Tokyo, Japan) was instilled in the fellow eye. Neither investigators nor volunteers had any information as to which eye received the nipradilol or placebo. Prior to instillation, both systemic and eye examinations, which included CDI measurements, were performed (baseline). The same examination was repeated 2 hr after the first instillation and at 1 week after the twice-daily instillations.

 As we evaluated the tendency of nipradilol on normal eyes, we designed next study for NTG eyes. A total of 10 patients with bilateral NTG who had not received any ocular medications for more than 4 weeks prior to evaluation (NTG group) were examined in the second part of the study. None of the patients had any history of intraocular surgery nor were receiving any systemic medication. NTG was defined as progressive glaucomatous optic nerve damage that occurred in the absence of either an increased IOP (less than 21 mmHg) or any other cause for optic nerve abnormalities. All systemic and ocular examinations, including the CDI measurements, were performed before the nipradilol instillation. Patients underwent the same examinations at 2 hr after the initial instillation and after 4 weeks of twice-daily instillations.

### 2.2. Systemic and Ocular Examination

Eye examinations, which included IOP measurements, heart rate (HR), and systemic blood pressure (BP), were measured for both the normal and NTG groups using a BP-203RVII (Colin, Tokyo, Japan). Ocular perfusion pressure (OPP) was calculated from the collected data using the following formula:

(1) $$\begin{array}{cc} \text{OPP} & =\frac{2}{3}\times [\text{diastolic}\;\text{BP}\;\\ & \;\;\;\;+\left(\frac{1}{3}\times \left(\text{systolic}\;\text{BP}-\text{diastolic}\;\text{BP}\right)\right)]\\ & \;-\text{IOP}. \end{array}$$

All CDI measurements were performed in the supine position using an LOGIQ 500 system (GE Yokogawa Medical Systems, Tokyo, Japan) equipped with a 7.5-MHz linear array transducer. Measurements were taken in the ophthalmic artery (OA), central retinal artery (CRA), temporal short posterior ciliary artery (TPCA), and nasal short posterior ciliary arteries (NPCAs). Using these images, we then calculated several parameters such as the peak systolic velocity (PSV), end-diastolic velocity (EDV), and resistance index (RI) for each vessel using the provided software. RI was calculated for each vessel using the following formula:

(2) $$\text{RI}=\frac{\left(\text{PSV}-\text{EDV}\right)}{\text{PSV}}.$$

### 2.3. Statistical Analysis

All data are expressed as the mean ± standard deviation. Values of each parameter before and after administration were compared using the Wilcoxon signed-rank sum test. An unpaired Student's *t*-test was used for comparison between the two groups. Values of *P* < 0.05 were considered statistically significant.

## 3. Results

### 3.1. Ocular Blood Flow after Nipradilol Administration in Normal Eyes

There were no significant differences between the nipradilol and placebo-treated eyes for the IOP and OPP baseline values. There were also no significant differences noted for the HR, BP, and OPP baseline values for the 2-hour and 1-week time points (Table 1). The mean IOP decreased in both the nipradilol- and placebo-treated eyes at 2 hr and at 1 week as compared to the baseline values.

 For the CDI examination, a large change in the retrobulbar hemodynamics was noted after the nipradilol administration (Table 2). PSV was significantly decreased in the OA at 2 hr (*P* = 0.0044). There was a significant increase in the EDV (*P* = 0.0365) along with a significant decrease of the RI (*P* = 0.0164) in the TPCA. At 1 week, there was a significant decrease of the RI in the OA (*P* = 0.0329), and the EDV increased significantly in the CRA (*P* = 0.0409). In addition, there was a tendency for the RI to decrease in the CRA and for the EDV to increase in the OA and TPCA. Conversely, there were no significant changes for any of these CDI values for the placebo-treated eye.

### 3.2. Ocular Blood Flow after Nipradilol Administration in NTG Eyes

As compared to the baseline values, there was a decrease in the mean IOP at 2 hr (*P* = 0.0004) and at 4 weeks (*P* = 0.0052) (Table 3). While there was also a significant decrease of the HR and OPP at 2 hr (*P* < 0.05) as compared to baseline, no change was noted at 4 weeks (Table 3).

 The CDI examination also found obvious changes in the retrobulbar hemodynamics after nipradilol administration in the NTG eyes compared to baseline. As seen in Table 4, there were significant decreases for the PSV in the OA (*P* = 0.0044), CRA (*P* = 0.0198), and TPCA (*P* = 0.0048) at 2 hr. The EDV was also significantly increased in the CRA (*P* = 0.0003), NPCA (*P* = 0.0064), and TPCA (*P* = 0.0001), and there was a significant decrease in the RI in the CRA (*P* = 0.0002), NPCA (*P* = 0.0023), and TPCA (*P* = 0.0007). At 4 weeks, the only significant increases were for the PSV in the NPCA (*P* = 0.0365) and for the EDV in the CRA (*P* = 0.0133). A significant decrease was noted for the RI in the CRA (*P* = 0.0010), NPCA (*P* = 0.0124), and TPCA (*P* = 0.0040).

## 4. Discussion

Using the CDI system, this study demonstrated the effect of topical nipradilol on retrobulbar hemodynamics in normal and NTG eyes. Topical nipradilol markedly decreased the peripheral vascular resistance in normal eyes as shown by the EDV or RI in the TPCA at 2 hr and in the OA at 1 week. As we observed the tendency that nipradilol affects normal eyes, we also evaluated its effects on NTG eyes. At both 2 h and 4 weeks in the NTG eyes, there was a decrease in the peripheral vascular resistance in the CRA, TPCA, and NPCA. These results indicate that topical 0.25% nipradilol increases the retrobulbar blood flow in both normal and NTG eyes.

 Recently, there have been many new instruments and techniques developed for noninvasive measurements of the ocular circulation, such as the laser Doppler method, scanning laser Doppler flowmetry, and the laser speckle method [13], which can evaluate the circulation of the visible vessels on the fundus. The CDI system used in the present study can detect blood flow by overlaying data on an ultrasonic B-mode image and then displaying the real-time direction and velocity of the blood flow of the invisible retrobulbar vessels using the Doppler shift frequency as the real-time blood flow velocity. Furthermore, peripheral vascular resistance corresponds to the resistance of the ciliary vascular bed in the optic disc and is represented by the RI or EDV. This can be readily calculated using the highest and lowest blood flow velocities. Since peripheral vascular resistance reflects the peripheral arterial circulation, it is now widely used as an index of therapeutic results [14]. Due to the high reproducibility of the CDI system, it is particularly appropriate for blood flow measurements in the short posterior ciliary arteries supplying blood to the cribriform plate [15]. The short posterior ciliary arteries like NPCA and TPCA supply blood to the anterior region of the cribriform plate directly or through the peripapillary choroidal blood flow, while the CRA supplies blood to the disc surface and posterior region of the cribriform plate. Thus, as compared to the OA and CRA, the NPCA and TPCA are more closely associated with the circulation in this region. During the development of glaucomatous optic nerve damage, the cribriform plate of the optic disc is the main region that is impaired [16]. Therefore, circulation disorders involving these vessels may be related to the damages seen in NTG. In this study, we demonstrated an improvement of circulation in the TPCA/NPCA for the normal and NTG eyes after nipradilol administration.

 Nipradilol has been shown to generate nitric oxide (NO) [17–20]. It is also well known that NO plays a crucial role in the ocular circulation or visual transduction [21]. When taken together, it is possible that nipradilol induces NO production, which subsequently can lead to an improvement in the retrobulbar hemodynamics. This suggests that nipradilol might have an influence on the anterior region of the cribriform plate and thus may be able to prevent glaucomatous damage in the eye through its donative production of NO.

 Recently, many studies have examined the use of oral medications to improve not only the general circulation but also the local ocular circulation. For example, oral calcium antagonists work not only as a hypotensive drug but also act by improving the peripheral vascular resistance of the short posterior ciliary arteries, thereby protecting the visual field [22]. While these findings demonstrate the importance of changes in the circulation, the use of systemic medications can sometimes lead to severe general side effects. Conversely, topical eye drops can reduce these side effects and provide much safer treatments. Eye drops generally penetrate the eye via several routes, such as the conjunctive, cornea, and sclera. By being able to penetrate the eye from the periocular tissue, nipradilol is able specifically to target the retina [23]. Our findings for the retrobulbar arteries indicated that nipradilol reached the posterior portion of the eye and not only had an effect on the retina via the sclera, but also was able to directly alter the retrobulbar hemodynamics. This mechanism of action makes it possible to improve the blood circulation of the optic nerve and protect the eye from glaucomatous damage.

 While our findings are promising, the small patient sample number and the short observation period did not allow us to determine how long nipradilol is effective or whether it could prevent glaucomatous visual field damage. Further investigations on the improvement of retrobulbar hemodynamics after long-term use of topical 0.25% nipradilol will need to be undertaken.

 In summary, short-term observation indicated that topical nipradilol increased ocular blood flow by decreasing resistance in the peripheral blood vessels of both normal and NTG eyes. The use of topical drugs makes it possible to influence retrobulbar hemodynamics through local penetration and affect the short posterior ciliary arteries. These findings suggest that the use of topical 0.25% nipradilol may be advantageous as compared to other antiglaucoma eye drops.

## Figures and Tables

**Table 1:** Systemic and ocular parameters before and after topical application of nipradilol or placebo in controls.

Parameter	Nipradilol			Placebo		
	Before	2 hrs (*P*)	1 week (*P*)	Before	2 hrs (*P*)	1 week (*P*)
Intraocular pressure (mmHg)	14.3 ± 1.6	11.5 ± 2.6 (0.0047)*	11.2 ± 2.2 (0.0071)*	13.7 ± 1.3	12.5 ± 2.5 (0.1212)	11.9 ± 1.9 (0.0144)*
Blood pressure						
Systolic (mmHg)	125.7 ± 10.2	126.2 ± 7.1 (0.6542)	123.2 ± 7.6 (0.7550)	125.7 ± 10.2	126.2 ± 7.1 (0.6542)	123.2 ± 7.6 (0.7550)
Diastolic (mmHg)	73.1 ± 6.6	73.2 ± 6.4 (0.9591)	72.6 ± 6.1 (0.8584)	73.1 ± 6.6	73.2 ± 6.4 (0.9591)	72.6 ± 6.1 (0.8584)
Heart rate (beats/min)	69.8 ± 9.7	67.2 ± 8.6 (0.1967)	66.4 ± 8.8 (0.1379)	69.8 ± 9.7	67.2 ± 8.6 (0.1967)	66.4 ± 8.8 (0.1379)
Ocular perfusion pressure (mmHg)	46.2 ± 5.2	49.1 ± 4.2 (0.0505)	48.5 ± 3.9 (0.2477)	46.7 ± 5.0	48.1 ± 4.6 (0.3281)	47.7 ± 3.9 (0.4496)

Wilcoxon signed-rank sum test.

Values are presented as mean ± standard deviation.

(*P*)* < 0.05.

**Table 2:** Results of color Doppler imaging in normal volunteers.

Parameter	Nipradilo1			Placebo		
	Before	2 hrs (*P*)	1 week (*P*)	Before	2 hrs (*P*)	1 week (*P*)
Ophthalmic artery						
PSV (cm/sec)	35.8 ± 8.4	31.8 ± 8.6 (0.0044)*	34.8 ± 8.6 (0.4769)	32.3 ± 10.8	31.8 ± 8.5 (0.6379)	31.6 ± 10.1 (0.5303)
EDV (cm/sec)	8.03 ± 2.40	7.52 ± 2.55 (0.4236)	8.76 ± 2.69 (0.0969)	8.17 ± 3.95	7.79 ± 2.13 (0.8753)	8.30 ± 2.89 (0.9063)
Resistance index	0.776 ± 0.035	0.764 ± 0.046 (0.2858)	0.749 ± 0.033 (0.0329*)	0.753 ± 0.065	0.753 ± 0.037 (0.7836)	0.738 ± 0.036 (0.3078)
Central retinal artery						
PSV (cm/sec)	11.3 ± 3.1	11.4 ± 2.4 (0.9999)	12.0 ± 2.5 (0.1095)	11.9 ± 2.4	12.4 ± 2.2 (0.3739)	12.6 ± 2.4 (0.1823)
EDV (cm/sec)	4.18 ± 1.06	4.28 ± 0.91 (0.5937)	4.67 ± 0.92 (0.0409*)	4.53 ± 1.27	4.38 ± 1.03 (0.8240)	4.73 ± 0.94 (0.8589)
Resistance index	0.627 ± 0.038	0.622 ± 0.051 (0.4145)	0.606 ± 0.058 (0.1414)	0.623 ± 0.052	0.646 ± 0.059 (0.2662)	0.622 ± 0.057 (0.9999)
NPCA						
PSV (cm/sec)	8.13 ± 1.85	8.36 ± 1.60 (0.4769)	8.02 ± 1.46 (0.8589)	9.43 ± 1.69	9.79 ± 1.22 (0.4243)	9.08 ± 0.89 (0.3743)
EDV (cm/sec)	3.43 ± 1.10	3.53 ± 0.78 (0.6833)	3.33 ± 0.85 (0.8588)	3.73 ± 0.81	4.06 ± 0.53 (0.2408)	3.66 ± 0.57 (0.5746)
Resistance index	0.584 ± 0.059	0.579 ± 0.041 (0.4496)	0.584 ± 0.067 (0.9999)	0.605 ± 0.051	0.582 ± 0.056 (0.2477)	0.596 ± 0.054 (0.3863)
TPCA						
PSV (cm/sec)	10.2 ± 2.8	10.0 ± 3.1 (0.7895)	10.6 ± 2.9 (0.5337)	11.3 ± 3.0	11.0 ± 3.1 (0.7213)	10.9 ± 2.3 (0.2475)
EDV (cm/sec)	3.95 ± 1.27	4.25 ± 1.45 (0.0365)*	4.36 ± 1.68 (0.4236)	4.53 ± 1.36	4.72 ± 1.74 (0.4496)	4.44 ± 1.35 (0.2662)
Resistance index	0.614 ± 0.060	0.578 ± 0.057 (0.0164)*	0.600 ± 0.065 (0.2477)	0.597 ± 0.050	0.584 ± 0.043 (0.3739)	0.592 ± 0.074 (0.9292)

Values are presented as mean ± standard deviation. PSV: peak systolic velocity, EDV: end-diastolic velocity, NPCA: nasal short posterior ciliary artery, TPCA: temporal short posterior ciliary artery.

Wilcoxon signed-rank sum test.

(*P*)* < 0.05.

**Table 3:** Systemic and ocular parameters before and after topical application of nipradilol in normal-tension glaucoma.

Parameter	Before	2 hrs (*P*)	4 weeks (*P*)
Intraocular pressure (mmHg)	15.3 ± 2.2	13.7 ± 2.2 (0.0004)*	14.0 ± 1.3 (0.0052)*
Blood pressure			
Systolic (mmHg)	130.0 ± 19.2	131.0 ± 21.7 (0.6456)	129.0 ± 19.8 (0.7206)
Diastolic (mmHg)	78.7 ± 13.4	81.2 ± 11.8 (0.1363)	77.7 ± 11.6 (0.5139)
Heart rate (beats/min)	71.4 ± 11.9	65.7 ± 11.7 (0.0067)*	68.1 ± 13.6 (0.3316)
Ocular perfusion pressure (mmHg)	48.6 ± 9.2	51.5 ± 8.8 (0.0002)*	49.2 ± 8.8 (0.4443)

Wilcoxon signed-rank sum test.

Values are presented as mean ± standard deviation.

(*P*)* < 0.05.

**Table 4:** Results of color Doppler imaging in normal tension glaucoma with topical nipradilol.

Parameter	Before	2 hrs (*P* value)	4 week (*P* value)
Ophthalmic artery			
PSV (cm/sec)	28.4 ± 10.8	24.7 ± 9.3 (0.0028)*	26.0 ± 9.0 (0.0871)
EDV (cm/sec)	7.71 ± 3.73	7.07 ± 3.26 (0.0859)	7.44 ± 3.67 (0.3490)
Resistance index	0.725 ± 0.084	0.712 ± 0.071 (0.4552)	0.721 ± 0.058 (0.7022)
Central retinal artery			
PSV (cm/sec)	9.89 ± 3.31	10.9 ± 4.4 (0.0198)*	10.3 ± 4.0 (0.3317)
EDV (cm/sec)	2.88 ± 1.17	3.50 ± 1.43 (0.0003)*	3.25 ± 1.35 (0.0133)*
Resistance index	0.711 ± 0.046	0.676 ± 0.035 (0.0002)*	0.683 ± 0.041 (0.0010)*
NPCA			
PSV (cm/sec)	9.03 ± 3.23	9.52 ± 4.31 (0.3134)	7.67 ± 2.43 (0.0365)*
EDV (cm/sec)	3.18 ± 1.40	3.77 ± 1.97 (0.0064)*	2.92 ± 0.94 (0.9108)
Resistance index	0.651 ± 0.061	0.607 ± 0.050 (0.0023)*	0.616 ± 0.052 (0.0124)*
TPCA			
PSV (cm/sec)	8.76 ± 2.53	10.2 ± 3.4 (0.0048)*	8.43 ± 2.24 (0.5256)
EDV (cm/sec)	2.95 ± 0.93	4.05 ± 1.43 (0.0001)*	3.25 ± 1.01 (0.1303)
Resistance index	0.662 ± 0.055	0.601 ± 0.048 (0.0007)*	0.615 ± 0.047 (0.0040)*

Values are presented as mean ± standard deviation. PSV: peak systolic velocity, EDV: end-diastolic velocity, NPCA: nasal short posterior ciliary artery, TPCA: temporal short posterior ciliary artery. Wilcoxon signed-rank sum test.

(*P*)* < 0.05.
